# Galectin-3-Mediated Glial Crosstalk Drives Oligodendrocyte Differentiation and (Re)myelination

**DOI:** 10.3389/fncel.2018.00297

**Published:** 2018-09-12

**Authors:** Laura Thomas, Laura Andrea Pasquini

**Affiliations:** ^1^Department of Biological Chemistry, School of Pharmacy and Biochemistry, University of Buenos Aires, Buenos Aires, Argentina; ^2^Institute of Chemistry and Biological Physicochemistry (IQUIFIB), National Scientific and Technical Research Council (CONICET), Buenos Aires, Argentina

**Keywords:** galectin-3, oligodedrocytes, myelination, CNS, remyelination, microglia, cytoskeleton, cuprizone

## Abstract

Galectin-3 (Gal-3) is the only chimeric protein in the galectin family. Gal-3 structure comprises unusual tandem repeats of proline and glycine-rich short stretches bound to a carbohydrate-recognition domain (CRD). The present review summarizes Gal-3 functions in the extracellular and intracellular space, its regulation and its internalization and secretion, with a focus on the current knowledge of Gal-3 role in central nervous system (CNS) health and disease, particularly oligodendrocyte (OLG) differentiation, myelination and remyelination in experimental models of multiple sclerosis (MS). During myelination, microglia-expressed Gal-3 promotes OLG differentiation by binding glycoconjugates present only on the cell surface of OLG precursor cells (OPC). During remyelination, microglia-expressed Gal-3 favors an M2 microglial phenotype, hence fostering myelin debris phagocytosis through TREM-2b phagocytic receptor and OLG differentiation. Gal-3 is necessary for myelin integrity and function, as evidenced by myelin ultrastructural and behavioral studies from *LGALS3^-^*^/^*^-^* mice. Mechanistically, Gal-3 enhances actin assembly and reduces Erk 1/2 activation, leading to early OLG branching. Gal-3 later induces Akt activation and increases MBP expression, promoting gelsolin release and actin disassembly and thus regulating OLG final differentiation. Altogether, findings indicate that Gal-3 mediates the glial crosstalk driving OLG differentiation and (re)myelination and may be regarded as a target in the design of future therapies for a variety of demyelinating diseases.

## Introduction

Galectins (Gals) constitute a 15-member family of β-galactoside-binding lectins which recognize *N*-acetyllactosamine and, despite lacking specific receptors, form multivalent complexes with cell surface glycoconjugates containing suitable oligosaccharides and trigger intracellular signals to regulate cell survival and differentiation (Rabinovich et al., 2007; Yang et al., 2008). Among Gals, Gal-1, -8, and -9 exert similar immunosuppressive and anti-inflammatory effects on the pathogenesis of demyelinating diseases such as Multiple Sclerosis (MS) and its experimental models (Anderson et al., 2007; Toscano et al., 2007; Starossom et al., 2012; Rinaldi et al., 2016; Pardo et al., 2017). In contrast, the role of Gal-3 seems to be more complex and even controversial.

In this context, the present review firstly summarizes Gal-3 structure, location in cells and tissues, extracellular and intracellular space functions, regulation, internalization and secretion. The manuscript then focuses on the current knowledge of Gal-3 role in central nervous system (CNS) health and disease, particularly regarding oligodendrocyte (OLG) differentiation, myelination and remyelination, and its potential as a target in the design of future therapies for a variety of demyelinating diseases.

### Gal-3 Structure

Gal-3 is a 25–35 KDa unique protein from the evolutionarily conserved family of Gals, which share a carbohydrate-recognition domain (CRD) and bind to β-galactoside-containing glycoconjugates. Gals are classified into three groups on the basis of their structural architecture: proto, chimera and tandem types, with Gal-3 being the only representative of the chimeric type (Barondes et al., 1994). Gal-3 has three structural domains: (a) the NH2 terminal domain containing serine phosphorylation sites Ser^6^ and Ser^12^, important for nuclear localization and oligomerization; (b) a proline and glycine-rich collagen-like sequence susceptible to metalloprotease (MMP) cleavage; and (c) a COOH terminal domain containing the CRD and the NWGR anti-death motif, highly conserved within the BH1 domain of the Bcl-2 protein family (Dumic et al., 2006; Funasaka et al., 2014). In the presence of ligand galactose, Gal-3 is able to form pentamers whose affinity increases when galactose binds other saccharides like *N*-acetyllactosamine (Hirabayashi et al., 2002; Ahmad et al., 2004).

### Gal-3 Tissue and Cellular Localization

Gal-3 is a ubiquitously distributed protein in adult tissue but has a tissue-time-dependent expression pattern throughout mouse embryogenesis (Poirier and Robertson, 1993; Fowlis et al., 1995; Van den Brule et al., 1997). In terms of cell populations, Gal-3 expression has been detected in fibroblasts, chondrocytes, osteoblasts, osteoclasts, keratinocytes, Schwann cells, gastric mucosa and endothelial cells from various tissues and organs (reviewed by Dumic et al., 2006). In addition to reports on neuronal and glial expression (Yoo et al., 2017), Gal-3 has been shown expressed *in vivo* by astrocytes in the subventricular zone (SVZ), being indispensable for cytoarchitecture maintenance but dispensable for apoptosis and proliferation (Comte et al., 2011). Gal-3 also maintains cell motility toward the olfactory bulb, possibly through EGFR phosphorylation modulation (Comte et al., 2011). Abundant evidence shows Gal-3 expression in cells committed to the immune response such as neutrophils, eosinophils, basophils, mast cells, Langerhans cells, dendritic cells, monocytes and macrophages from different tissues (Holíková et al., 2000; Jin et al., 2005; Chen et al., 2006; Sundblad et al., 2011; Novak et al., 2012; Ge et al., 2013; Wu et al., 2017; Brittoli et al., 2018). Even in cell types which do not normally express it such as lymphocytes, Gal-3 expression can be induced by various stimuli like T-cell receptor ligation, viral transactivating factors, and calcium ionophores (Hsu et al., 2009). Gal-3 is also expressed in several types of tumors, with expression intensity depending on tumor progression, invasiveness and metastatic potential (Danguy et al., 2002; reviewed in van den Brule et al., 2004). Regarding intracellular localization, Gal-3 is found in both cell cytoplasm and nucleus (Haudek et al., 2010) and is secreted to the extracellular space where it is often incorporated to the extracellular matrix (ECM) (Krześlak and Lipińska, 2004). Worth pointing out, Gal-3 functions are tightly dependent on localization.

### Gal-3 Functions

**FIGURE 1:**
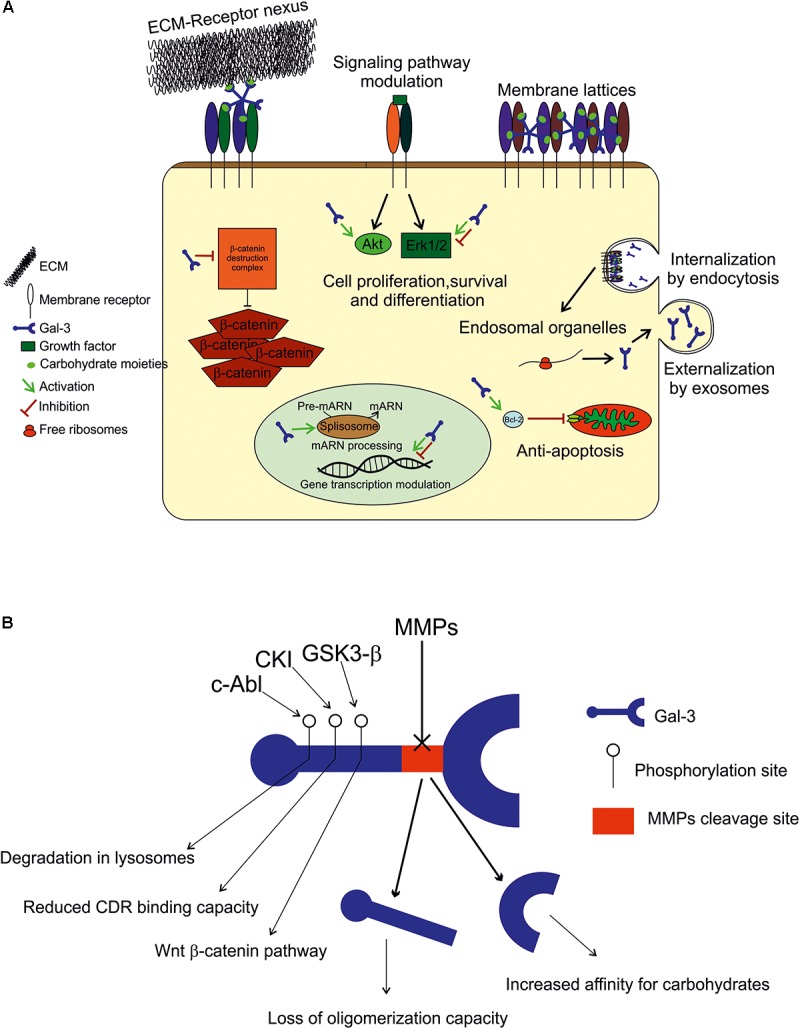
**(A)** *Extracellular space:* Gal-3 either binds to the ECM compounds (laminin, hensin, elastin, collagen IV, tenascin-C and -R, and integrin) to modulate cell adhesion, or interacts with plasmatic membrane receptors by binding to carbohydrate moieties in an autocrine or paracrine fashion to form membrane lattices and trigger intracellular events. *Intracellular space:* within the cell, Gal-3 is found in both cytoplasm and nucleus, and its binding appears to be mediated by protein-protein interactions. In the cytoplasm, it plays an anti-apoptotic role (Bcl-2), and modulates signaling pathways (Akt and in Erk 1/2) to promote or inhibit cell growth, proliferation and differentiation. In the nucleus, Gal-3 is crucial for pre-mRNA splicing (spliceosome incorporation) and to promote or repress transcription. *Internalization and secretion*: Gal-3 enters the cell by non-clathrin-mediated endocytosis and goes through endosomal organelles to be later sorted elsewhere or degraded. Gal-3 is secreted via a non-classical pathway and apparently through exosomes. **(B)** *Regulation:* Gal-3 activity is regulated by MMP2 and MMP9 cleavage (Ala62 to Tyr63), generating a 22 kDa whole CRD peptide (high affinity for carbohydrates) and a 9 kDa N-terminal peptide (oligomerization capacity). Also, Gal-3 is phosphorylated in Tyr residues by c-Abl kinase to promote its own degradation in lysosomes in Ser residues by casein kinase I to reduce its carbohydrate binding capacity, and by GSK3-β to regulate the Wnt-β-catenin pathway.

A summary of Gal-3 functions in the extracellular and intracellular space, its regulation and its internalization and secretion is provided in **Figure 1**.

#### Extracellular Space

As it lacks a secretion signal sequence, Gal-3 is secreted via a non-classical pathway (described in *Gal-3 secretion and internalization*). It is often incorporated to the ECM to modulate cell adhesion or interact with plasmatic membrane receptors by binding to carbohydrate moieties in an autocrine or paracrine fashion to form membrane lattices and trigger intracellular events. Gal-3 impact on cell adhesion and migration depends on its multivalent capacity, as it functions as a nexus between ECM and membrane receptors and can thus inhibit or potentiate these cell properties. Gal-3 binds laminin, hensin, elastin, collagen IV, tenascin-C and -R, and crosslinks with integrins, the major cell adhesion receptor modulators (reviewed by Dumic et al., 2006). Regarding membrane receptors, those having five or more glycosylated sites are preferable candidates for lattice formation, like TGFβR and EGFR, while those with fewer glycosylation sites are below the threshold. This scenario may change, however, when *N*-glycosylation is stimulated with UDP-GlcNAc (Lau et al., 2007; Nabi et al., 2015). Extracellular Gal-3 has been described mainly as an inflammatory mediator: for example, it is chemoattractant to monocytes, increases superoxide anion production in monocytes and neutrophils, increases apoptosis in T-cells and other cell types depending on the doses used, increases LPS responses and decreases T-cell receptor (TCR)-mediated signaling in T-cells, among other functions (Hsu et al., 2009; Dabelic et al., 2012; Xue et al., 2017; Uchino et al., 2018). Also, depending on cell types, extracellular Gal-3 can promote or inhibit cell proliferation and growth (Krugluger et al., 1997; Inohara et al., 1998; Diao et al., 2018; Zhou et al., 2018).

#### Intracellular Space

Within the cell, Gal-3 is distributed between the cytoplasm and the nucleus depending on cell types and conditions (Haudek et al., 2010). In the intracellular compartment most Gal-3 binding appears to be mediated by protein–protein interaction rather than protein-carbohydrate recognition. In the cytoplasm, it plays an anti-apoptotic role, as it interacts with Bcl-2, and it also modulates signaling pathways in order to promote or inhibit cell growth, proliferation and differentiation. It activates Akt (Kariya et al., 2018; Ruvolo et al., 2018), inhibits or activates Erk 1/2 (Alge-Priglinger et al., 2011; Mori et al., 2015; Zhang et al., 2017), and increases β-catenin levels (Shimura et al., 2004; Hu et al., 2015), among other functions, depending on the cell types evaluated. In the nucleus, Gal-3 is crucial for pre-mRNA splicing, as it is incorporated into spliceosomes by associating with the U1 small nuclear ribonucleoprotein (snRNP) complex (Patterson et al., 2002; Haudek et al., 2010). Gal-3 has also been shown to promote or repress the transcription of certain genes (Krugluger et al., 1997; Iacobini et al., 2017; Zhou et al., 2018).

### Gal-3 Regulation

Gal-3 is regulated mainly by degradation by MMPs, phosphorylation and space-time coordinated expression, as described above. Aminoacid sequence analyses have revealed that Gal-3 is cleaved by MMP2 and MMP9 between Ala^62^ and Tyr^63^, resulting in two peptides: a 22 kDa peptide containing the whole CRD and a 9 kDa N-terminal peptide (Gao et al., 2017). The CRD peptide retains its carbohydrate-binding activity with increased affinity for carbohydrates (Ochieng et al., 1994). However, Gal-3 capability to oligomerize is lost upon cleavage, which generates changes in Gal-3 functions such as the interaction with membrane receptors (Nabi et al., 2015). Therefore, Gal-3 cleavage by MMP is of high importance to determine its activity. Also worth noticing, Gal-3 induces the expression and secretion of MMP9 in melanoma cells, indicating a feedback loop for Gal-3 regulation (Mauris et al., 2014; Dange et al., 2015). Also, in other cell types, Gal-3 is known to be phosphorylated in Tyr residues by c-Abl kinase (Balan et al., 2010) to promote its own degradation in lysosomes (Li et al., 2010), in Ser residues by casein kinase I to reduce its carbohydrate binding capacity (Mazurek et al., 2000), and by GSK3-β to regulate the Wingless (Wnt)-β-catenin pathway (Song et al., 2009).

### Gal-3 Internalization and Secretion

It is known that Gal-3 is incorporated into the cell through non-clathrin-mediated endocytosis and goes through endosomal organelles to be later sorted elsewhere or degraded (Hönig et al., 2015; Nabi et al., 2015). Regarding externalization, Gal-3 lacks a signal for classical externalization and it was recently found in the lumen of apical secreted exosomes from epithelial cells. For this type of secretion, Gal-3 release depends on the endosomal sorting complex required for transport I, essential for cargo recruitment (Bänfer et al., 2018). This notion is of great relevance for further exploration, as exosomes are being increasingly described as a short and long-distance communication pathway between cells. However, the mechanisms, if any, through which Gal-3 can be released out of exosomes and into the extracellular space in a free form remain to be elucidated.

### Gal-3 Roles in Non-Cns Disease

Secreted Gal-3 is currently under evaluation as a biomarker of tumoral and fibrotic processes, as it has been found elevated in many cancers including solid tumors and blood cancers like leukemias and lymphomas (reviewed in Ruvolo, 2016). Gal-3 is thought to be produced by tumor microenvironment cells such as mesenchymal stromal cells and to play immunosuppressive functions, thus supporting metastasis (Ruvolo, 2016). Gal-3 also takes part in tumor cell adhesion, proliferation, differentiation, angiogenesis, and metastasis (Wang and Guo, 2016). In fibrotic processes, Gal-3 has been principally described in skin illnesses (McLeod et al., 2018) and liver and heart failure (Moon et al., 2018; Piek et al., 2018). In acute ischemic stroke, higher levels of serum Gal-3 have been recently associated with increased death risk (Wang et al., 2018). Likewise, it is extensively described in inflammatory processes modulating macrophage responses (de Oliveira et al., 2015; Mietto et al., 2015; Schnaar, 2016). In addition, Gal-3 seems to exert an inhibitory effect on LPS-induced inflammation, as increased inflammatory cytokine production is induced in both *LGALS3*^-/-^ macrophages and wild-type macrophages in which Gal-3 binding has been neutralizated through antibodies. This was also supported by an increase in *LGALS3*^-/-^ mice vulnerability to LPS, reflected by higher inflammatory cytokine and nitric oxide production (Li et al., 2008).

## Gal-3 in Healthy OLG Differentiation

**FIGURE 2:**
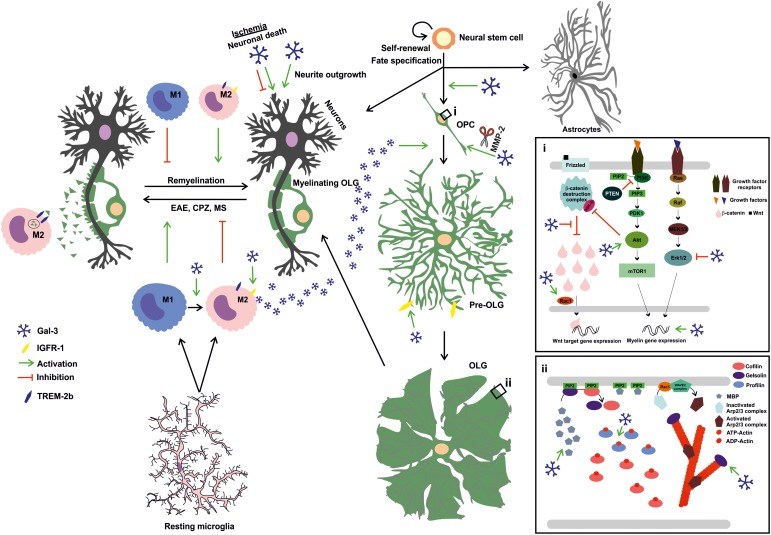
Extracellular Gal-3 drives OLG differentiation and (re)myelination: Gal-3 is expressed in microglial cells during remyelination, favoring an M2 microglial phenotype and, therefore, fostering myelin phagocytosis through TREM-2b phagocytic receptor and OLG differentiation probably by IGFR-1 pathway stimulation. In OPC (i), Gal-3 drives early process outgrowth through enhanced actin assembly and a decrease in Erk 1/2 activation, and later regulates OLG maturation (ii) by inducing Akt activation and inhibiting β-catenin degradation to increase MBP expression and promotes gelsolin release and actin cytoskeleton disassembly. Also, microglia-released Gal-3 induces OLG fate in neural stem cells. Finally, Gal-3 promotes neurite outgrowth but exerts both deleterious and protective effects on neuronal death in ischemic brain injury.

Having introduced the main features of Gal-3, the present review will now focus on the current knowledge of Gal-3 role in CNS myelination, remyelination and OLG differentiation (**Figure 2**).

### Gal-3 Expression and Regulation in OLG

Oligodendrocyte are resident CNS cells in charge of myelination, i.e., the physiological process of axon insulation allowing metabolic and trophic support for axons and providing a rapid saltatory conduction of action potentials (Bercury and Macklin, 2015). In terms of morphological and molecular stages, OLG are initially bipolar cells named oligodendrocyte progenitor cells (OPC) which proliferate and migrate, expressing molecular markers like PDGFRα and NG2. An intermediate stage represented by pre-OLG, more ramified, express CNPase, Olig 1 and O4, among others. These cells later form myelin membranes and express MBP, APC, and PLP, hence becoming the fully mature OLG capable of myelination (Nave and Werner, 2014; Snaidero and Simons, 2017).

Gal-3 mRNA expression has not yet been evaluated in OLG, which makes it still uncertain whether it is actually expressed in this cell type and whether it acts in an autocrine fashion to regulate OLG maturation. However, Gal-3 has been immunocytochemically detected in immature (PDGFRα, A2B5, and O4) and mature (O1, MBP, CNPase, PLP) OLG isolated from primary rat cultures, with higher levels in mature ones (Pasquini et al., 2011). Recent work also outlines Gal-3 occasional expression by Rip+ OLG in white matter from normal rat brains (Yoo et al., 2017). Gal-3 appears to be cleaved in OPC by MMP2 and then stabilized in mature OLG, which suggests variations in its biological activity during OLG differentiation (Pasquini et al., 2011).

### Gal-3 Drives OLG Maturation

As part of pioneering studies on the direct influence of Gal-3 on OLG maturation, in 2011 our group demonstrated that extracellular Gal-3 promotes OLG differentiation and may be expressed and secreted by microglia (Pasquini et al., 2011). Microglia are known to express Gal-3 in normal oligodendrogenesis (Ellison and de Vellis, 1995; Pasquini et al., 2011) and upon activation, promoting myelin debris phagocytosis through FcγR, CR3/MAC-1 and SRAI/II receptors and fostering remyelination upon injury or disease (Rotshenker et al., 2008). Work by Pasquini et al. (2011) has revealed that extracellular Gal-3 promotes dose-dependent OLG differentiation in close relationship with the glycoconjugates present in OPC cell surfaces. Glycosylation signature analysis has shown that OPC possess a permissive glycophenotype expressing the necessary carbohydrates for Gal-3 binding. In contrast, Gal-3 overexpression in the OLG N20.1 cell line produces no changes in MBP promoter activity, which indicates that changes in endogenous Gal-3 relative abundance do not affect OLG differentiation. Moreover, treatment with conditioned media from wild type (WT) microglia, but not *LGALS3*^-/-^ microglia, promotes OLG differentiation, suggesting that microglia express the Gal-3 necessary to induce OLG maturation. In addition, using neurosphere cultures, our group has also proven Gal-3 to promote cell commitment toward the OLG lineage (Pasquini et al., 2011).

### Gal-3 Accelerates OLG Differentiation: Actin Cytoskeleton Dynamics Modulation

Although studies on the processes controlling signaling pathways and cytoskeleton dynamics in OLG have rendered contrasting results, recent thorough reports proposed a two-step model for actin dynamics in OLG: first, pro-assembly dynamics until OLG are fully differentiated and, second, a switch to pro-disassembly dynamics to start myelination (Nawaz et al., 2015; Zuchero et al., 2015). This is driven by interplay between MBP and actin disassembly proteins like cofilin-1 and gelsolin, both sequestered and hence inactivated by phosphatidylinositol 4,5-bisphosphate (PIP2) on the plasma membrane. When MBP is expressed as OLG reach their final maturational state, MBP competes for PIP2 binding, and gelsolin and cofilin-1 are displaced from their binding to the inositol and activated to drive actin disassembly (Zuchero et al., 2015). Further work has demonstrated that cytoplasmic gelsolin is crucial for OPC differentiation and is a downstream effector of anti-LINGO1 blocking antibody. This study also shows that cytoplasmic gelsolin expression drives OPC differentiation and increases as OLG mature, indicating that cytoskeleton severing proteins are crucial for morphological changes required for OLG differentiation (Shao et al., 2017). Gal-3 effects on the actin cytoskeleton appear to be cell type-dependent, driving cytoskeleton disassembly dependent on Erk 1/2 inactivation in retinal pigment epithelium (RPE) cells (Alge-Priglinger et al., 2011), while promoting lamellipodia formation in epithelial cells (Saravanan et al., 2009).

In the B16F10 murine melanoma cell line, cells plated over Gal-3 as substrate have shown increased actin turnover and lamellipodia formation on the cell edges, and an increase in Rac1 activation (More et al., 2016). Indeed, our most recent work first unveiled the role of extracellular Gal-3 in OLG cytoskeleton dynamics (Thomas and Pasquini, 2018). Our group demonstrated that extracellular Gal-3 modulates actin cytoskeleton dynamics in primary rat OLG cultures in two steps: first, Gal-3 drives actin polymerization dynamics and then switches to depolymerization dynamics, which may be thought of as an accelerated version of the process described by Zuchero et al. (2015). These changes are accompanied by an increase in gelsolin and MBP expression, generating actin disassembly in a carbohydrate-dependent manner as OLG mature. Moreover, Gal-3 induces an increase in the expression of actin ADT-ATP exchanger profilin-1 and activated small GTPase Rac-1, further supporting its crucial role in actin dynamics. Likewise, Gal-3 accelerates OLG differentiation as compared to vehicle-treated cells, as supported by the increase observed in CNPase+ cells and in the area and expression of MBP (Thomas and Pasquini, 2018).

### Gal-3 Accelerates OLG Differentiation: Underlying Signaling Pathways

Conserved signaling pathways in OLG like Erk 1/2, β-catenin and Akt play an important, though controversial, role in OLG maturation.

#### Erk 1/2

Some *in vitro* reports have shown Erk 1/2 inhibition to diminish OLG maturation (Fyffe-Maricich et al., 2011; Guardiola-Diaz et al., 2012; Xiao et al., 2012), while other studies *in vivo* and *in vitro* have reported no changes upon Erk 1/2 inactivation in OPC differentiation (Ishii et al., 2012, 2013; Xiao et al., 2012). Also, inhibiting Erk 1/2 decreases OPC proliferation in response to growth factors (Bhat and Zhang, 1996; Kumar et al., 1998; Baron et al., 2000; Bansal et al., 2003; Cui and Almazan, 2007; Frederick et al., 2007). Erk 1/2 has been proposed to contribute to the passage from OPC to pre-OLG (Narayanan et al., 2009; Guardiola-Diaz et al., 2012), while other reports claim that it promotes the passage from pre-OLG to mature OLG (Tyler et al., 2009; Bercury et al., 2014). A thorough revision of the involvement of Erk 1/2 signaling in CNS myelination has been recently published by Gonsalvez et al. (2016). In addition, work by More et al. in 2016 revealed that tumor cells plated on Gal-3 show a time-dependent decrease in Erk 1/2 phosphorylation. Similarly, extracellular Gal-3 decreases Erk 1/2 activation in RPE cells (Alge-Priglinger et al., 2011) and in keratinocytes, generating anti-apoptotic events (Larsen et al., 2011). Our recent work indicates that Gal-3 inhibits Erk 1/2 in a carbohydrate-dependent manner, thus contributing to the promotion of MBP and gelsolin expression and subsequent OLG maturation (Thomas and Pasquini, 2018).

#### Akt/mTOR

The role of the Akt/mTOR pathway is less controversial than that of Erk 1/2, as it is known to improve myelination both *in vitro* and *in vivo*. However, as with Erk 1/2, the timing of Akt action has not fully elucidated yet, although available evidence suggests that it favors the passage from pre-OLG to OLG, in a close crosstalk with Erk 1/2 (reviewed in Gaesser and Fyffe-Maricich, 2016). *In vivo*, Akt overexpression as well as PTEN inhibition generates a hypomyelinated phenotype (Flores et al., 2008; Narayanan et al., 2009). mTOR is known to promote lipid synthesis by activating SREBPS, which is of high importance in promoting myelin synthesis (reviewed in Figlia et al., 2018). Further support for the pro-myelinating role of mTOR has been obtained through the use of rapamycin, mTOR inhibitor, revealing that mTOR is crucial for OPC differentiation, and myelin protein and mRNA synthesis (Tyler et al., 2009, 2011; Guardiola-Diaz et al., 2012).

Several authors have demonstrated that Gal-3 activates Akt signaling. In human cutaneous squamous cell carcinoma tissues, Gal-3 binds to β4-integrin and activates the Akt axis (Kariya et al., 2018). Also, in mammary epithelial tumor cells, extracellular Gal-3 activates PI3K, upstream kinase in the Akt pathway (Lagana et al., 2006). In tumor cell line B16F10, Akt phosphorylation increases with time in cells plated on Gal-3 as substrate (More et al., 2016). In line with these results, our work proves that Gal-3 augments Akt activation also in a carbohydrate-dependent fashion, which leads to an increase in MBP and gelsolin expression, driving OPC differentiation (Thomas and Pasquini, 2018).

#### β-catenin

β-catenin is a protein degraded by the proteasome when phosphorylated by a destruction complex in the cytoplasm, an event which requires absence of Wnt ligand binding to Frizzled receptor. When binding occurs, β-catenin is not degraded and can translocate to the nucleus where it acts as a transcription factor modulating cellular functioning. In OLG, some authors describe this pathway as an OLG differentiation inhibitor (Fancy et al., 2009; Feigenson et al., 2009; Ye et al., 2009), while others describe it as promoting OLG differentiation (Kalani et al., 2008; Tawk et al., 2011; Ortega et al., 2013). These differences probably respond to the conditions used and the timing of evaluation.

In colon cancer cells, Gal-3 has been shown to drive β-catenin accumulation by regulating GSK-3β phosphorylation –a member of β-catenin destruction complex– via the PI3K/Akt pathway (Song et al., 2009). A similar conclusion has been reached in gastric cancer cells, where absence of Gal-3 diminishes β-catenin levels (Kim et al., 2010). Our results show that Gal-3 augments β-catenin expression in mature OLG, concomitantly with an increase in MBP expression and Akt activation. β-catenin levels decrease when Akt is inhibited, further supporting the close connection between these pathways (Thomas and Pasquini, 2018).

### Gal-3 Drives Axon Myelination and Neurite Outgrowth

To investigate Gal-3 *in vivo* role during the myelination process, our group has analyzed Gal-3 expression at postnatal day 5 (P5), 10 (P10), 15 (P15), and 20 (P20) using transgenic mice expressing the enhanced green fluorescent protein (EGFP) driven by the promoter of oligodendroglial protein CNPase (CNP–EGFP). Gal-3 expression showed substantial changes during white matter development, with high expression levels at P5 and a reduction upon myelin development. In agreement, high levels of Gal-3 were found at P5 in microglial cells localized in the CC and cingulum, fairly close to CNPase+ cells. Interestingly, confocal microscopy showed some CNPase+ cells with maturen OLG-like morphology to colocalize with Gal-3. On the other hand, Gal-3 was detected at low levels in astrocytes at P10 and P15, and at high levels in the SVZ at all ages evaluated. This work also revealed a critical role for Gal-3 during OLG myelination, as reflected by the morphological changes observed in the myelin of *LGALS3*^-/-^ mice. These animals presented hypomyelination in the striatum and CC, as revealed by electron microscopic morphometric analysis. These myelin defects were deeper in 8-week-old relative to 4-week-old mice. Also, myelin showed lesser integrity and abnormal compaction compared to WT mice. Furthermore, *LGALS3*^-/-^ mice exhibited substantial alterations in behavior with diminished levels of innate anxiety than their WT littermates (Pasquini et al., 2011).

Interestingly, substratum-bound Gal-3 had been previously shown to promote outgrowth of neurites from dorsal root ganglia explants (Pesheva et al., 1998) and neurite stabilization in the cerebellum (Mahoney et al., 2000). In this context, further work needs to be carried out in order to elucidate Gal-3 role in myelination *in vivo* and whether this neurite outgrowth and stabilization could influence OLG myelin formation capacity.

## Gal-3 in CNS Disease

The main features of Gal-3 in CNS diseases are summarized in **Figure 2**.

### Inflammation and Hypoxic-Ischemic Injury

In CNS disease, Gal-3 has been attributed both deleterious and protective effects, being mainly expressed by activated microglia and upregulated by inflammatory stimuli (Umekawa et al., 2015). In a focal cerebral ischemia model in mice, Gal-3 was found to mediate microglial activation and proliferation and to be preferentially expressed by a subgroup of proliferating IGF-1-expressing microglia. Tunicamycin-mediated inhibition of *N*-glycosylation diminishes IGF-1-induced microglial mitogenic response, while coimmunoprecipitation assays have revealed Gal-3 binding to IGF-receptor 1, which suggests that interactions between Gal-3 and N-linked glycans present in growth factor receptors are mediators of IGF-receptor 1 (IGF-R1) signaling (Lalancette-Hébert, 2007; Rotshenker, 2009; Lalancette-Hébert et al., 2012). Moreover, an increase in Gal-3 expression is observed in several ischemic models, associated to an improvement in neurologic function, a reduction in neuronal cell death, angiogenesis, proliferation of neuronal precursors, and a type 2 T cell immune bias (Ohtaki et al., 2008; Yan et al., 2009). These findings suggest that Gal-3 may act as an important modulator of the brain inflammatory response with high neuroprotective potential. However, in other models of hypoxic-ischemic (HI) injury, Gal-3 has been shown to exert deleterious effects. For instance, *LGALS3*^-/-^ mice are protected from neuronal death in the hippocampus and striatum, and a reduction in Gal-3 is observed during delayed neuronal death induced by hypothermia or antiapoptotic agents in the CA1 region (Doverhag et al., 2010; Satoh et al., 2011a,b; Hisamatsu et al., 2016). Further studies have also shown that the increase in Gal-3 expression observed in ischemia is blocked when antiapoptotic molecule Bis is downregulated, generating less vulnerable hippocampal neurons (Cho et al., 2012). In addition to the deleterious effects, Gal-3 has been suggested as a target for the treatment of post-stroke gastrointestinal complications, as it is released after stroke and triggers central and peripheral enteric neuronal loss through a TLR4-mediated mechanism involving TAK1 and AMPK (Cheng et al., 2016). Finally, recent studies conclude that Gal-3 is necessary for angiogenesis after ischemia but does not affect apoptosis, infarct size, microglial, and astrocyte activation/proliferation, SVZ neurogenesis or migration to lesion (Young et al., 2014; Xu et al., 2017).

### Amyotrophic Lateral Sclerosis, Alzheimer’s Disease, Parkinson’s Disease and Prions

In amyotrophic lateral sclerosis (ALS), advanced glycation end-products (AGE) are a source of inflammation and oxidative injury. Gal-3 acts as an AGE receptor and leads them to lysosome degradation and removal (Pricci, 2000). In line with this, Gal-3 counteracts neuroinflammation, as its deletion leads to heightened neurodegeneration in ALS owing to AGE accumulation (Lerman, 2012). Moreover, Gal-3 has emerged as a key biomarker candidate on account of its differential expression in ALS mouse model SOD1 (G93A) and spinal cord tissue and cerebrospinal fluid from ALS patients (Zhou et al., 2010). Its expression is also increased in microglia accompanied by osteopontin and vascular endothelial growth factor (VEGF), concomitantly with low expression of TNFα, IL-6, brain-derived neurotrophic factor (BDNF) and arginase-1 from ALS rat model SOD1 (G93A) (Nikodemova et al., 2014). Gal-3 might also be postulated as a potential biomarker for Alzheimer’s disease (AD), as AD patients exhibit significantly higher levels of serum Gal-3 (Wang et al., 2015). In Parkinson’s disease (PD), extracellular α-synuclein-aggregate-induced inflammation can be reduced by Gal-3 inhibition, which suggests therapeutic potential for Gal-3 (Boza-Serrano et al., 2014). In addition, microarray analysis of substantia nigra in a PD animal model has shown the temporal expression profiles of 4 candidate genes implicated in neuroglial activation and functional maturation, i.e., Gal-3, Heat shock protein 27, Lipocalin 2 and Tissue inhibitory metalloproteinase 1 (Choy et al., 2015). In prion-infected brain tissue, Gal-3 has been found to exert harmful effects (Riemer et al., 2004; Mok et al., 2006, 2007), as its expression in activated microglia/macrophages correlates with abnormal prion protein accumulation (Jin et al., 2007).

### MS and Its Experimental Models

Oligodendroglial injury leads to demyelination, which is followed by a regenerative response consisting in the formation of new myelin sheaths –a process called remyelination (Franklin, 2002; Franklin and Ffrench-Constant, 2017). This process has been described in animal models and in human demyelinating diseases such as MS (Prineas et al., 1993; Patrikios et al., 2006; Patani et al., 2007). MS course varies considerably among patients, although the most frequent presentation consists of recurring clinical symptoms followed by total or partial recovery, namely the classic relapsing-remitting form of MS. After 10–15 years of disease, symptoms become progressive in up to 50% of untreated patients and lead to clinical deterioration for several years, a stage referred to as secondary progressive MS. In about 15% of MS patients, however, disease progression is relentless as from onset, in what constitutes primary progressive MS (Bjartmar et al., 2001; McFarland and Martin, 2007; Tallantyre et al., 2010). The progressive stage is in part due to incomplete remyelination, which produces the loss of axonal metabolic support provided by myelin and concomitant axonal and neural degeneration, which lead to the progressive disability observed in the later stages of MS (Nave, 2010; Franklin et al., 2012).

Sera from patients with secondary progressive MS has been recently shown to present auto-antibodies against Gal-3, which may be responsible for blood brain barrier progressive damage (Nishihara et al., 2017). These authors determined that membrane-bound Gal-3 in human brain microvascular endothelial cells (BMEC) is a target for auto-antibodies present in secondary progressive MS serum but not for healthy donors or patients with other CNS illnesses; downregulation of Gal-3 in these cells causes an increase in the expression of intracellular adhesion molecule-1 (ICAM-1) and phospho-NFκB p65, both molecules described as responsible for leukocyte leakage to the CNS (Dietrich, 2002). These effects are also observed when BMEC are incubated with secondary progressive MS serum but prevented when Gal-3 is downregulated in BMEC or when secondary progressive MS serum is depleted from anti-Gal-3-auto-antibodies. These results hint at Gal-3 and anti-Gal-3 antibodies as therapeutic targets to prevent blood brain barrier damage.

Further work has described the effect of cerebrospinal fluid (CSF) from patients with primary progressive MS or relapsing remitting MS in the morphology and transcriptional response of treated OPC (Haines et al., 2015). The results obtained indicate that OPC treated with primary progressive MS CSF present a significantly more ramified morphology than control or relapsing remitting MS CSF, accompanied by a pro-differentiating transcriptome: downregulation of PDGFRα and LINGO1 genes and upregulation of MAG gene. However, this transcriptome is different from that present in normal OPC differentiation. Interestingly, this work reports *LGALS3* gene upregulation only in primary progressive MS CFS-treated OPC and establishes a link between Gal-3 upregulation and increased OPC branching. Gal-3 upregulation has also been observed in post-mortem human brain tissues with primary progressive MS.

Taken together, these findings indicate that Gal-3 is a positive target for OLG differentiation in human MS tissue and drives the downregulation of ICAM-1 in BMEC, mediating a protective effect on the blood–brain barrier. However, the presence of anti-Gal-3 auto-antibodies is responsible for blood brain barrier damage, a negative effect which might be counteracted through neutralizating therapy.

Current evidence has proposed exosomes as possible biomarkers and therapeutic agents in CNS disease. Most importantly, as therapeutic agents, exosomes display some advantages, as they can deliver cargo to other cells, rapidly pass the brain–blood barrier and render low immunogenicity (Chen and Chopp, 2018; reviewed in Osier et al., 2018). As described before in this review, Gal-3 is known to be excreted through exosomes, which opens doors for the evaluation of the therapeutic potential and biomarker capacity of the presence of Gal-3 in exosomes.

Even if experimental models fail to replicate MS in its full complexity and heterogeneity, they have succeeded in developing various treatments for MS patients. Several well-established experimental demyelination models include those mediated by immunity, virus and toxins. The most widely used animal model of CNS demyelination is experimental autoimmune encephalomyelitis (EAE), in which mice are immunized with myelin oligodendroglial glycoprotein. EAE constitutes the most widely exploited model and is especially useful to study the autoimmunity aspects in MS pathology. However, numerous therapeutic agents displaying beneficial effects in this model have proven poorly or not beneficial at all in the treatment of MS. *LGALS3^-/-^* mice display clearly diminished CNS macrophage infiltration during EAE, which leads to lesser disease severity (Jiang et al., 2009). These findings suggest a central role of Gal-3 in promoting inflammation by leukocyte recruitment. In addition, Gal-3 is induced in several cell types involved in damaged axon and cell debris removal and axon regeneration and remyelination, which hints at neuroprotective role of Gal-3 in EAE mice (Itabashi et al., 2018).

Virus-induced demyelination models support the hypothesis that some environmental factors, such as viral infections, are involved in MS and may be actually triggering the disease. MS-induced inflammation may decrease SVZ cell proliferation and thus hinder repair. Gal-3 expression increases in active human MS lesions (Stancic et al., 2011), in periventricular regions in human MS and after murine TMEV infection (James et al., 2016), whereas Gal-3 loss reduces the number of immune cells in the SVZ and restores proliferation in a viral model of MS (James et al., 2016).

Another increasingly used and more recently described demyelination model consists in the administration of cuprizone (CPZ) as part of the diet of young adult mice, which produces massive demyelination through pathogenic T cell-independent mechanisms, with different areas specifically affected in the CNS such as the CC (Suzuki and Kikkawa, 1969; Blakemore, 1973; Ludwin, 1978; Matsushima and Morell, 2001). CPZ-induced demyelination is known to involve the recruitment of resident microglia, while peripheral macrophage infiltration seems to be still controversial (McMahon et al., 2002; Mildner et al., 2007; Lampron et al., 2015). The CPZ model then offers the advantage of investigating CNS processes leading to remyelination independently of peripheral immune system contribution, as the model keeps the blood brain barrier intact (Bakker and Ludwin, 1987; Kondo et al., 1987).

Previous studies have described four different lesion subtypes in MS: pattern-1 and -2 lesions are thought to be autoimmune response-mediated, while pattern-3 and -4 lesions are regarded as primary oligodendrogliapathy (Lucchinetti et al., 2000). The first two types are experimentally simulated by the EAE model, while the second two are mimicked by toxic models such as cuprizone (CPZ) or lysolecithin (LPC) administration. Although the established animal models have their advantages and disadvantages, no model fully replicates the stages of MS and they are actually complementary.

#### Gal-3-in the CPZ Model

##### Gal-3-induced microglial response modulation during demyelination

Phagocytosis of myelin debris by microglia in CPZ demyelination is concomitant with an increase in phagocytic receptor TREM-2b expression (Voß et al., 2011), which seems to be key for remyelination to occur, as oligodendroglial differentiation can be hampered by myelin debris (Kotter et al., 2006). Strikingly, myelin phagocytosis relies on CR3/MAC-1 and SRAI/II, in turn regulated by Gal-3-dependent activation of PI3K; therefore, myelin phagocytosis by *LGALS3*^-/-^ microglia is usually deficient (Rotshenker et al., 2008).

Our group has studied Gal-3 involvement in the demyelination/remyelination process using the CPZ model (Hoyos et al., 2014, 2016), in which 8-week-old *LGAL3*^-/-^ and WT mice were fed a diet containing 0.2% CPZ w/w for 6 weeks to evaluate demyelination, followed by two more weeks on a CPZ-free diet to assess remyelination. Our results have shown that CPZ-induced demyelination follows a similar course up to the 5th week of treatment in 8-week-old *LGALS3*^-/-^ and WT mice, as demonstrated by MBP immunostaining and electronic microscopy assessment. The OPC generated in response to CPZ demyelination in *LGALS3*^-/-^ mice display reduced branching, which reflects diminished differentiation. These findings are in agreement with our results showing Gal-3 ability to induce OLG differentiation and especially those proving that conditioned media from Gal-3-expressing microglia promote OLG differentiation, as different from conditioned media from *LGALS3*^-/-^ microglia (Pasquini et al., 2011). WT mice exhibit spontaneous remyelination in the 5th week of CPZ treatment, even if the CPZ diet is kept in place until the 6th week. In contrast, *LGALS3*^-/-^ mice lack this ability and suffer steady demyelination up to the 6th week with noticeable astroglial activation. Colocalization studies have shown that Gal-3 expression is upregulated in microglia but not in astrocytes during CPZ-induced demyelination. Interestingly, only WT mice display activated microglia with ED1 (CD68) expression and TREM-2b upregulation during CPZ-induced demyelination, unlike CPZ-treated *LGALS3*^-/-^ mice, which display more numerous microglia with activated caspase-3. These findings support Gal-3 as a modulator of the microglial response to favor the onset of remyelination and OLG differentiation (Hoyos et al., 2014).

As mentioned above, TREM-2b plays a key role in myelin debris phagocytosis. TREM-2b loss-of-function causes a hereditary disease called polycystic lipomembranous osteodysplasia with sclerosing leukoencephalopathy (PLOSL), or Nasu–Hakola disease (Hakola, 1972; Nasu et al., 1973; Paloneva et al., 2001), which presents progressive presenile dementia and sclerosing leukoencephalopathy driven by lower microglial phagocytic activity (Paloneva et al., 2002). Microglial TREM-2b expression is upregulated in inflammatory and chronic phases of EAE and produces disease exacerbation when blocked during the effector phase (Piccio et al., 2007). Intravenous inoculation of TREM-2-transduced myeloid cells induces EAE amelioration accompanied by increased myelin phagocytosis (Takahashi et al., 2007). In parallel, CPZ-induced demyelination has also shown a robust increase in microglial phagocytic activity associated with the upregulation of TREM-2b and CD200R, and including TNF-α production (Voß et al., 2011). Interestingly, our results show *LGALS3*^-/-^ mice inability to upregulate TREM-2b or increase TNF-α production but their capacity to significantly reduce CD200R as a response to CPZ toxicity, which suggests that Gal-3 absence alters the microglial response against demyelination.

Our results showing that Gal-3-deficient microglial cells fail to induce ED1 expression are in accordance with those by Lalancette-Hébert et al. (2012), obtained in a unilateral transient focal cerebral ischemia model. However, and in contrast with such results, *LGALS3*^-/-^ mice submitted to CPZ-induced demyelination exhibit higher microglial proliferation and apoptosis, as evidenced by their increased activation of caspase-3, which may respond to the well-established Gal-3 anti-apoptotic role (Yang et al., 1996).

Evidence has demonstrated that chemokine ligand 2 (CCL2 or MCP1) could be involved in MS pathology, as its neutralization with anti-CCL2 significantly reduces EAE relapsing severity (Karpus and Kennedy, 1997). Our results show that CCL2 mRNA levels remain significantly higher until the 5th week in the CC of CPZ-treated *LGALS3*^-/-^. In contrast, WT mice display transiently higher CCL2 mRNA levels in the 1st week of CPZ treatment with a recovery to control values in the 2nd week, which indicates that CCL2 mediates early microglial activation. M2 polarization of macrophages is accompanied by a reduction in inflammatory cytokine IL-1β, TNF-α, and CCL2 (Liu et al., 2013). Taken together, these results could indicate that the absence of Gal-3 prevents microglial cells from shifting to an M2 phenotype. Indeed, the fact that M2-cell-conditioned media enhance OLG differentiation *in vitro* and that M2 cell depletion impairs OLG differentiation *in vivo* may indicate that M2 cell polarization is a key factor for efficient remyelination (Miron et al., 2013), and microglial expression of Gal-3 may hence favor the onset of remyelination, either by inducing an M2 phenotype or exerting a direct effect on OLG differentiation.

##### Gal-3 in MMP regulation during remyelination

Together with endogenous tissue inhibitors, the MMP family of zinc-dependent endopeptidases is a key player in tissue remodeling. MMPs can also induce myelin proteins degradation *in vitro* (Chandler et al., 1995, 1996; Shiryaev et al., 2009; Hansmann et al., 2012), and have been involved in postnatal myelination, myelin maintenance, and remyelination (Ulrich et al., 2006; Skuljec et al., 2011; Hansmann et al., 2012). Also, MMP-3 can mediate mature OLG apoptosis and microglial activation with production of microglial inflammatory cytokines and thus exacerbation of neural cell degeneration (Kim and Joh, 2006). Our immunohistological studies have shown an increase in MMP-3 expression and a decrease in CD45+, TNFα+, and TREM-2b+ cells during remyelination only in WT mice, with no changes in *LGALS3*^-/-^ mice during demyelination or remyelination. Ultrastructural studies carried out by electron microscopy after remyelination revealed collapsed axons with a defective myelin wrap in CC of *LGALS3*^-/-^ mice but no relevant myelin wrap disruption in sections obtained from WT mice. Worth highlighting, a blockade in OPC differentiation has been proposed as a possible cause for remyelination failure in demyelinating diseases (Franklin and Kotter, 2008; Ulrich et al., 2008). Therefore, *LGALS3*^-/-^ mice incomplete remyelination may partly respond to a failure in OPC differentiation. These results suggest that Gal-3 impacts remyelination through mechanisms including the tuning of microglial cells, the modulation of MMP activity and the induction of OLG differentiation.

##### Gal-3 role in behavioral alterations

CPZ-fed mice exhibit weight loss and motor and behavioral deficits (Franco-Pons et al., 2007; Xu et al., 2009). In the case of long-term CPZ exposure, rodents exhibit behavioral alterations which may be thought to recapitulate psychiatric disorders (Gregg et al., 2009; Xu et al., 2009). Neuroleptic treatment has succeeded in ameliorating some of these symptoms (Zhang et al., 2008; Xu et al., 2010, 2011), which suggests that long-term CPZ exposure may replicate features associated with MS and other white matter disorders. In this line, work by our group has shown diminished anxiety behavior in *LGALS3*^-/-^ mice, similar to that observed in early CPZ-induced demyelination (Pasquini et al., 2011). Interestingly, hypertensive rats used as a spontaneous model of attention deficit hyperactivity disorder exhibit lower expression of Gal-3 in the brain prefrontal cortex (Wu et al., 2010). Accordingly, the lack of Gal-3 and alterations in myelin structure in *LGALS3*^-/-^ mice induces behavioral alterations such as lower anxiety levels and spatial working memory impairment. Also, CPZ impact on behavior is observed earlier in *LGALS3*^-/-^ mice, probably due to early mature OLG depletion (Hesse et al., 2010; Buschmann et al., 2012).

In our remyelination studies, a decrease in anxiety persisted both in WT and *LGALS3*^-/-^ mice after 2-week recovery, as evidenced by higher rates of entries and more time spent in open arms in the plus maze test. An increase was also observed in locomotor activity counts and the number of total arm entries. In contrast, spatial working memory showed a recovery regarding their untreated counterparts. These data are in agreement with previous studies (Franco-Pons et al., 2007; Xu et al., 2009; Stancic et al., 2012) and suggest that some behavioral aspects remain altered even when histological analyses evidence fiber remyelination and seem to be irreversible even 2 weeks after CPZ withdrawal.

## Conclusion

Taken together, these reports show that Gal-3 is essential for microglial polarization following CNS injury, while the differences observed in Gal-3 effects on different injuries probably respond to time- and context-dependent factors. Rahimian et al. (2018) have recently discussed the possible molecular mechanisms of neuroprotection mediated by Gal-3. Firstly, oligomerized Gal-3 molecules may crosslink to IGFR upon binding to their glycans on the surface and delay their removal by endocytosis, which results in prolonged microglial mitogenic signaling (Partridge et al., 2004; Lalancette-Hébert et al., 2012). This proposed mechanism could also act as a mediator of the beneficial effects of Gal-3 on OLG differentiation and (re)myelination, as IGF-1 produced by microglia inhibits OLG apoptosis during CPZ-induced demyelination and thereby promotes remyelination (Mason et al., 2000) and acts directly on OLG and myelination, as shown using OLG lineage-specific IGF-1R^-/-^ mice (Zeger et al., 2007). Additionally, Gal-3 might mediate alternative microglial polarization induced by IL-4, as its expression and release are stimulated by alternative activation of macrophages with IL-4, which then binds and crosslinks CD98 on macrophages (Mackinnon, 2012). This could also explain the effect of Gal-3 observed on cell fate decisions toward the oligodendroglial lineage, as IL-4-activated microglia favor oligodendrogenesis, whereas IFN-gamma-activated microglia favor neurogenesis. The effect induced by IL-4-activated microglia is also mediated, at least in part, by IGF-I (Butovsky et al., 2006). In addition, Gal-3 promotes the proliferation of cultured neural progenitors and its inhibition decreases the proliferative response of the SVZ after brain ischemia (Yan et al., 2009).

In particular, our results have shown that Gal-3 expression in microglial cells during CPZ-induced demyelination and upon the onset of remyelination favors an M2 microglial phenotype and MMP activity modulation, leading to OLG differentiation. Furthermore, Gal-3 enhances actin assembly and reduces Erk 1/2 activation, thus driving early OLG branching. Gal-3 later induces Akt activation and higher MBP expression, which in turn promote gelsolin release and actin cytoskeleton disassembly, hence regulating OLG maturation. On the whole, our studies indicate that Gal-3 could be considered a novel extracellular signal which drives OLG differentiation and (re)myelination through glial cell communication and therefore a target in the design of future therapies for a variety of demyelinating diseases.

## Author Contributions

LT designed the figures. LT and LP wrote and designed the manuscript. LP supported manuscript submission.

## Conflict of Interest Statement

The authors declare that the research was conducted in the absence of any commercial or financial relationships that could be construed as a potential conflict of interest.

## Abbreviations

APC: adenomatous polyposis coli
CC: corpus callosum
CNPase: 2′, 3′-Cyclic-nucleotide 3′-phosphodiesterase
EGFR: epidermal growth factor receptor
GFAP: glial fibrillary acidic protein
IFN: interferon
IGF-1: insulin-like growth factor-1
LPS: lipopolysaccharide
MBP: myelin basic protein
mTOR: mammalian target of rapamycin complex
NG2: neural/glial antigen 2
Olig: oligodendrocyte transcription factor
PDGFRα: platelet-derived growth factor receptor α
PLP: myelin proteolipid protein
TGF: transforming growth factor
TNF: tumor necrosis factor
TREM-2: triggering receptor expressed on myeloid cells-2
